# Lower Expression of Ndfip1 Is Associated With Alzheimer Disease Pathogenesis Through Decreasing DMT1 Degradation and Increasing Iron Influx

**DOI:** 10.3389/fnagi.2018.00165

**Published:** 2018-06-08

**Authors:** Juan Tian, Wei Zheng, Xin-Lu Li, Yuan-Hong Cui, Zhan-You Wang

**Affiliations:** ^1^Institute of Health Sciences, Key Laboratory of Medical Cell Biology of Ministry of Education, China Medical University, Shenyang, China; ^2^Department of Histology and Embryology, Jinzhou Medical University, Jinzhou, China; ^3^Science and Technology Innovation System Construction Service Center of Liaoning Province, Shenyang, China

**Keywords:** Alzheimer’s disease, β-amyloid, Nedd4 family interacting protein 1 (Ndfip1), divalent metal transporter 1 (DMT1), iron metabolism

## Abstract

We have previously reported that high expression of divalent metal transporter 1 (DMT1) plays a crucial role in iron dyshomeostasis and β-amyloid (Aβ) peptide generation in the brain of Alzheimer’s disease (AD). Recent studies have shown that Nedd4 family interacting protein 1 (Ndfip1) can degrade DMT1 through ubiquitination pathway and reduce the accumulation of intracellular iron. The present study aims to evaluate whether Ndfip1 is involved in AD pathogenesis through mediating DMT1 degradation and iron metabolism. β-amyloid precursor protein/presenilin 1 (APP/PS1) transgenic mouse and Ndfip1 transfected SH-SY5Y cells were used in this study. Immunohistochemistry and Western blot were performed to examine the distribution and expression levels of Ndfip1 and DMT1. In addition, ELISA and calcein fluorescence were carried out for analyzing the levels of Aβ peptide and iron influx, respectively. The results showed that Ndfip1 immunoreactivity was decreased in the cortex and hippocampus of APP/PS1 mice, compared with wild type (WT) controls. Colocalization of Ndfip1 and Aβ within senile plaques could be observed. Immunoblot analyses showed that low expression of Ndfip1 and high expression of DMT1 proteins were detected in APP/PS1 mouse brain, compared with age-matched WT animals. Overexpression of Ndfip1 down-regulated DMT1 expression, and reduced iron influx and Aβ secretion in SH-SY5Y cells. Further, overexpressed Ndfip1 significantly attenuated iron-induced cell damage in Ndfip1 transfected cells. The present study suggests that lower expression of Ndfip1 might be associated with the pathogenesis of AD, through decreasing DMT1 degradation and increasing iron accumulation in the brain.

## Introduction

Alzheimer’s disease (AD) is a neurodegenerative disease characterized by progressive cognitive disorder in the elderly. The typical neuropathological changes in AD brain are β-amyloid (Aβ) peptide deposition, tau hyperphosphorylation and neuronal loss. Mounting evidence has demonstrated that brain iron is abnormally elevated in AD brain (Goodman, 1953; Smith et al., 1997; Liu et al., 2006; Yoshida, 2016; Adlard and Bush, 2018; Peters et al., 2018). Iron overload may lead to oxidative stress, Aβ and tau protein aggregation, as well as cell death (Mantyh et al., 1993; Yamamoto et al., 2002; Liu et al., 2011; Telling et al., 2017; Tripathi et al., 2017; Maher, 2018). Therefore, to clarify the mechanism underlying brain iron dyshomeostasis is helpful to understand the pathogenesis and prevention of AD.

Transmembrane transport of iron ions is essential for maintaining cellular iron homeostasis. Divalent metal transporter 1 (DMT1) is a widely expressed mammalian transmembrane metal-ion transporter, which is responsible for the uptake of a broad range of divalent metal ions, including iron ion (Fleming et al., 1997; Gunshin et al., 1997; Tandy et al., 2000; Nevo and Nelson, 2004; Duck et al., 2017; Wolff et al., 2018). The pathological increase in DMT1 levels has been found in neurodegenerative disorders, such as Parkinson’s disease (PD; Salazar et al., 2008; Zhang et al., 2009, 2017; Jiang et al., 2010; Xu et al., 2010; Du et al., 2016) and AD (Zheng et al., 2009; Xie et al., 2012). Mutation of DMT1 gene could protect mice against MPTP-induced dopaminergic neuronal death, through mediating cellular iron metabolism (Salazar et al., 2008). We have reported that DMT1 is colocalized with Aβ in senile plaques of postmortem human AD brain, and the protein levels of DMT1 are significantly elevated in the brain of β-amyloid precursor protein/presenilin 1 (APP/PS1) transgenic mice compared with wild type (WT) mice (Zheng et al., 2009). Further, *in vitro* studies have demonstrated that silencing of endogenous DMT1, not only reduces iron influx, but also leads to reductions of APP expression and Aβ secretion (Zheng et al., 2009). These suggest that changes in DMT1 expression may contribute to the neuropathogenesis of AD. However, why DMT1 is highly expressed in AD brain remains to be elucidated.

Nedd4 family interacting protein 1 (Ndfip1), also known as Nedd4 WW-domain-binding protein 5 (N4WBP5), plays a role in neuroprotection, through mediating ubiquitination of target proteins in neuronal injury (Howitt et al., 2009; Goh et al., 2014; Low et al., 2015). Interestingly, DMT1 is one of the Ndfip1 target proteins, and Ndfip1 can degrade DMT1 protein through ubiquitination pathway (Foot et al., 2008; Howitt et al., 2009; Garrick et al., 2012). Therefore, it is reasonable to speculate that changes in Ndfip1 expression may contribute to the degradation of DMT1 protein and subsequent accumulation of iron in the progression of AD.

In the present study, we aimed to analyze the distribution and expression level of Ndfip1 protein in APP/PS1 transgenic mouse brain. Furthermore, using Ndfip1 transfected SH-SY5Y cells, we examined the possible role of Ndfip1 in DMT1 degradation, iron influx, Aβ secretion, as well as in iron-induced cell damage.

## Materials and Methods

### Animals and Tissue Preparation

Male APP/PS1 double transgenic mice expressing a chimeric mouse/human Swedish mutation amyloid precursor protein (Mo/HuAPP695swe) and a mutant human presenilin 1 (PSEN1-dE9; Jankowsky et al., 2001), and WT C57BL/6 mice were obtained originally from the Jackson Laboratory (West Grove, PA, USA). Throughout the experiments, mice were kept in a controlled environment of 22–25°C, 40%–60% relative humidity, 12-h light/12-h dark cycle, with standard diet and distilled water available *ad libitum*. Nine-month-old mice were deeply anesthetized with sodium pentobarbital (50 mg/kg, intraperitoneally) and decapitated. The brains were immediately removed and split in half on an ice-clod board. Half of the brain was paraffin-embedded and 6-μm thick sections were prepared for morphological assessment. The other half of the brain was stored at −80°C for biochemical assays. This study was carried out in accordance with the recommendations of “Laboratory Animals-Guideline of welfare and ethics, The Ethics Committee for Medical Laboratory Animals of China Medical University.” The protocol was approved by The Ethics Committee for Medical Laboratory Animals of China Medical University.

### Cell Culture and Transfection

Human neuroblastoma SH-SY5Y cells stably over-expressing human APPsw or empty vector (*neo*) pCLNCXv.2 were made using lipofectamine 2000 (Invitrogen Inc., USA) and selected by G418 resistance, as reported previously (Zhang et al., 2006a,b; Zheng et al., 2009; Wang et al., 2011). The cells were grown in DMEM supplemented with 10% heat-inactivated fetal bovine serum (Gibco, USA), 100 IU/ml penicillin, and 100 μg/ml streptomycin at 37°C in a humidified incubator containing 5% CO_2_ air. cDNA of human Ndfip1 (GeneBank™ accession number NM_030571) was amplified from human brain cDNA by polymerase chain reaction (PCR) and subcloned into the vector pCMV-MCS (Genechem, China). APPsw cells were transiently transfected with pCMV-MCS- Ndfip1 for 24, 48 and 72 h using lipofectamine 2000.

### Assessment of Cell Viability

Cell viability was measured in 96-well plates by quantitative colorimetric assay with MTT (Denizot and Lang, 1986; Zheng et al., 2009; Wang et al., 2011). Briefly, at the indicated time after treatments, cells were continued incubated with medium containing 500 μg/ml MTT at 37°C for 3 h. Then the MTT solution was removed and treated with dimethyl sulfoxide (DMSO) to dissolve the colored formazan crystal. The absorbance at 595 nm of each aliquot was determined using a microplate reader (TECAN, Switzerland). Cell viability was expressed as the ratio of the signal obtained from treated cultures and control cultures.

### Calcein Loading of the Cells and Iron Transport Assay

Ferrous iron influx into cells was determined by quenching of calcein fluorescence. After transfected with pCMV-MCS-Ndfip1 and vector control, cells were loaded with 0.5 μM calcein AM (Dojindo Laboratories, Japan) for 30 min at 37°C according to a method described previously (Ci et al., 2003). Then excess calcein AM on the cell surface was removed by several washes with phosphate-buffered saline (PBS, pH 7.4). Just before measurement, 500 μl calcein-loaded cell suspension was added to the cuvette. After collecting the initial baseline of fluorescence intensity, ferrous sulfate (100 μM) was added to the cuvette. Then the fluorescence was continuously recorded in every second for 30 min with an F-4500 Fluorescene Spectrophotometer equipped with a stirring cuvette holder (Hitachi, Japan; λex of 490 nm, λem of 515 nm, 37°C). Data were normalized to the steady-state (baseline) values.

### Quantitation of Aβ1–42 Using Sandwich ELISA

The supernatants of medium were collected and a protease inhibitor cocktail (2.5 mM EDTA, 10 μM leupeptin, 1 μM peptastin and 1 mM phenylmethylsulfonyl fluoride) was added. Then the conditional medium supernatants were concentrated (10×) using a Freeze Vacuum Dryer (MARTIN CHRIST, Germany) and stored at −80°C for Sandwich ELISA. Aβ1–42 level was assayed with human Aβ 1–42 Colorimetric Immunoassay Kit (Invitrogen, USA), according to the manufacturer’s instruction. The absorbance was recorded at 450 nm with a microplate reader (TECAN).

### Immunohistochemistry and Confocal Laser Scanning Microscopy

Routine avidin-biotinylated complex (ABC) immunohistochemical staining was used to analyze the distribution of Aβ and Ndfip1 in APP/PS1 mouse brain. Briefly, paraffin sections were dewaxed in xylene, rehydrated through a series of decreasing concentrations of ethanol, and treated in 0.1 M Tris-HCl buffer (TBS, pH 7.4) containing 3% hydrogen peroxide (H_2_O_2_) for 10 min to reduce endogenous peroxidase activities. After rinsing with Tris-buffered saline, sections were boiled in TEG buffer for 5 min in a microwave oven. The sections were then rinsed, treated with 5% bovine serum albumin for 1 h, and subsequently incubated overnight with rabbit anti-Ndfip1 antibody (1:150, Sigma) at 4°C. After several rinses, the sections were incubated with biotinylated goat anti-rabbit IgG (1:200) for 1 h at room temperature, followed by amplification with streptavidin peroxidase for 1 h. The sections were then rinsed and stained with 0.025% 3,3-diaminobenzidine plus 0.033% H_2_O_2_ for 1 min. Some sections were counterstained with hematoxylin. Finally, the sections were observed and photographed using a light microscope equipped with a digital camera (Olympus, Tokyo, Japan). Control sections were incubated with identical solutions but without primary antibody.

For immunofluorescent staining and confocal microscopic analysis, sections or culture cells were pre-incubated with normal donkey serum (NDS, 1:20, Jackson ImmunoResearch Laboratory) for 1 h, and then incubated overnight in a mixture of primary antibodies, mouse anti-Aβ (1:500, Sigma) and rabbit anti-Ndfip1 (1:150). After several rinses, the sections or culture cell were incubated for 2 h with a mixture of secondary antibodies, FITC-conjugated donkey anti-mouse IgG (1:50), and Texas Red-conjugated donkey anti-rabbit IgG (1:50). To assess nonspecific staining, control sections were incubated with normal serum instead of primary antibodies. After rinsing with PBS, the sections and culture cells were mounted using an anti-fading mounting medium and examined with a confocal laser scanning microscope (SP2, Leica). Excitation filters for FITC (488 nm) and Texas-Red (568 nm) were selected. Images were collected and processed using an Adobe Photoshop program.

### Western Blot Analysis

Tissue homogenates of the mouse brains and lysates of culture cells were centrifuged at 12,000 rpm for 30 min at 4°C, and quantified for total proteins using the UV 1700 PharmaSpec ultraviolet spectrophotometer (Shimadzu, Japan). Proteins (60 μg) of each sample were separated on 10% sodium dodecyl sulfate polyacrylamide gels (SDS-PAGE) and transferred to polyvinylidene difluoride (PVDF) membranes (Millipore, CA, USA) using an electronasfer device (45 V, 15 h). After blocking in 5% fat-free milk in TBS containing 0.1% Tween-20 for 1 h, the membranes were then incubated with the following primary antibodies: anti-DMT1-IRE (1:2000, Alpha Diagnostic, San Antonio, TX, USA), anti-DMT1-nonIRE (1:1000, Alpha Diagnostic, San Antonio, TX, USA), anti-Ndfip1 (1:2000), anti-APP695 (1:4000, Chemicon, USA), and anti-GAPDH (1:10,000, KC-5G5, Kang Chen, China) for 2 h at room temperature. Then the membranes were washed and incubated with horseradish peroxidase-conjugated second antibody (1:5000, Santa Cruz, CA, USA) for 2 h at room temperature. Immunoreactive bands were visualized by an enhanced chemiluminescence kit (Pierce, Appleton, WI, USA) and ChemDocTM XRS with Quantity One™ software (BioRad, USA). Blots were repeated at least three times for every condition. The band intensities were quantified by Image-pro Plus 6.0 analysis software (Media Cybernetics, Rockville, MD, USA).

### Real Time PCR

Total RNA of cells was extracted using Trizol Reagent (Invitrogen, Carlsbad, CA, USA) according to the manufacturer’s protocol. A total of 5 μg RNA was reverse transcribed to cDNA using the reverse-transcription system kit (Promega, Madison, WI, USA) in the following reaction conditions: 37°C for 15 min followed by 85°C for 5 s. For quantitative PCR, each specific gene product was amplified with SYBR Green PCR Master mix (Applied Biosystems Inc., Carlsbad, CA, USA) by an Applied Biosystems Inc., 7300 Sequence Detection System. Fifty nanograms templates of cDNA were added to 20 μl reaction mixture. Each cDNA sample was prepared in triplicates. RT-PCR cycling conditions included pre-incubation at 50°C for 2 min, DNA polymerase activation by 35 cycles at 95°C for 5 min, followed by 40 cycles of denaturing at 95°C for 30 s and annealing and extension at 58°C for 30 s. The sequences of Ndfip1 and GAPDH genes were obtained from GenBank database, and specific primers were designed over an exon-exon junction with Primer Premier 5.0: Ndfip1: forward, 5′-CCAGCTGAGGATAGGAAACG-3′ and reverse, 5′-GGCATCTTCCGAACTT TTGC-3′; GAPDH: forward, 5′-GGATTTGGTCGTATTGGG-3′ and reverse, 5′-TCGCTCCTGGAAGATGG-3. Relative mRNA expression levels were calculated using cycle time (Ct) values normalized to the expression of GAPDH. Data were analyzed using Advanced Relative Quantification Software (Roche) and relative differences were expressed as a percentage relative to control.

### Statistics

All values are expressed as the mean ± SEM. One-way analysis of variance (ANOVA) or Student’s *t*-test was used for evaluation of differences among more than three groups or for the evaluation of differences between two groups, respectively. Results were reported to be statistically significant for values of *P* < 0.05 and highly statistically significant for values of *P* < 0.01.

## Results

### Low Expression of Ndfip1 Protein in APP/PS1 Transgenic Mouse Brain

**Figure 1:**
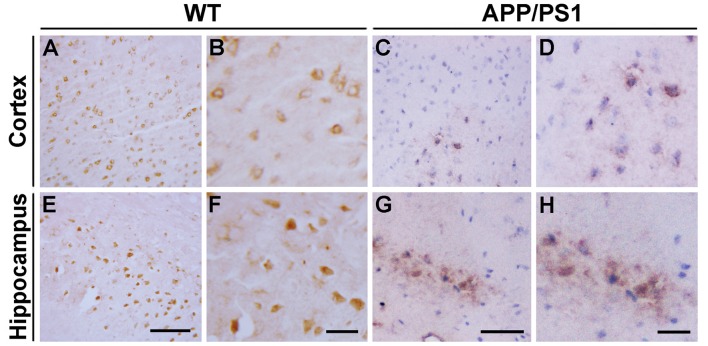
Immunohistochemical images showing the distribution of Nedd4 family interacting protein 1 (Ndfip1) in the cortex **(A–D)** and hippocampus **(E–H)** in wild type (WT) and β-amyloid precursor protein/presenilin 1 (APP/PS1) transgenic mice at 9 months of age. Scale bars = 200 μm.

We first analyzed the distribution of Ndfip1 protein in APP/PS1 transgenic mouse brain. Immunohistochemical results showed that Ndfip1 immunoreactive products were predominantly located in neuronal cell body. Compared with WT mice, APP/PS1 mice showed a decreased immunoreactivity of Ndfip1 in the cerebral cortex and hippocampal neurons (Figure 1).

**Figure 2:**
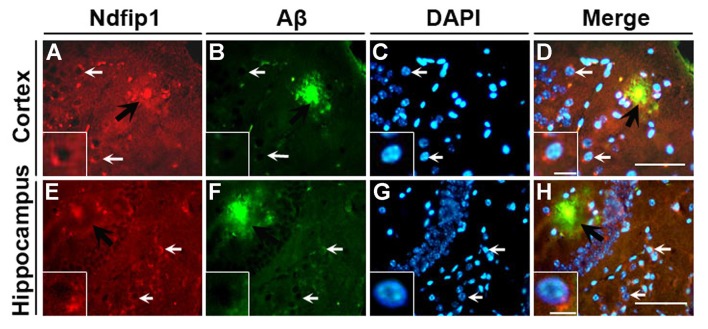
Colocalization of Ndfip1 and Aβ in APP/PS1 transgenic mouse brain. Cryostat sections from 9-month old APP/PS1 transgenic mouse brain were stained with Ndfip1 **(A,E)** and Aβ **(B,F)** antibodies. The nucleus was stained with DAPI **(C,G)**. Black arrows indicate the localization Ndfip1 in Aβ plaques in the cortex **(A–D)** and hippocampus **(E–H)**. White arrows and insets indicate Ndfip1-postive neurons. Scale bars = 250 μm **(A–H)**; 50 μm (insets).

We have previously reported that the Ndfip1 target protein, DMT1, could be found in amyloid plaques in the brains of human AD postmortem and APP/PS1 transgenic mouse (Zheng et al., 2009). Therefore, we used confocal laser scanning microscopy to detect whether Ndfip1 was also located in Aβ plaques. Immunofluorescence double-labeling with Ndfip1 and Aβ showed that Ndfip1 was co-localized with Aβ in senile plaques in APP/PS1 mouse brain (Figure 2), and there is no positive immunoreaction in the negative control sections.

**Figure 3:**
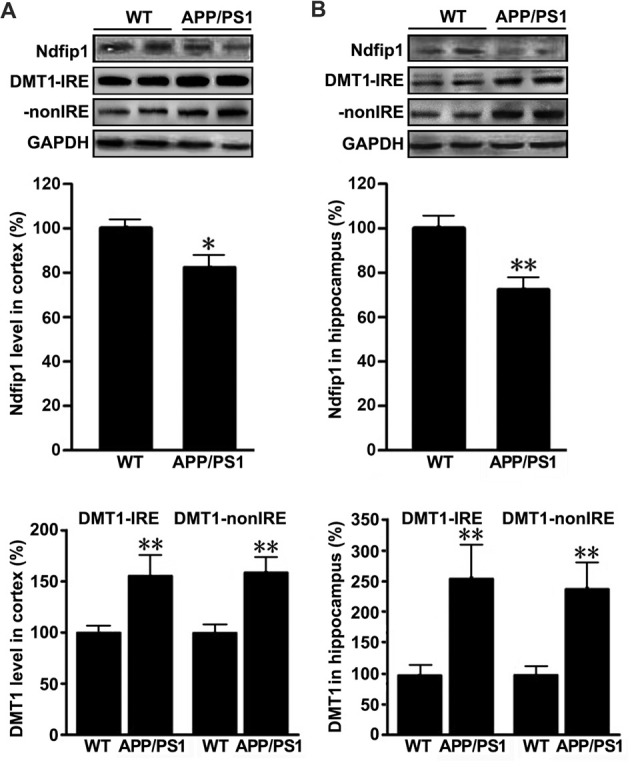
Expression levels of Ndfip1 and divalent metal transporter 1 (DMT1) in APP/PS1 transgenic mouse brain. Immunoblotting analyses revealed that Ndfip1 protein levels were significantly decreased in the cortex **(A)** and hippocampus **(B)** of APP/PS1 mice, compared with WT controls. On the contrary, the protein levels of both DMT1-IRE and DMT1-nonIRE were significantly elevated in APP/PS1 mouse brain (*n* = 6; *P < 0.05, **P < 0.01).

To quantify the expression levels of Ndfip1 and divalent metal transporter 1 (DMT1) in the brains of APP/PS1 transgenic mice and WT control mice, we extracted proteins from the cortex and hippocampus. Immunoblot analyses for Ndfip1 revealed a major band at 26 kDa, matching the predicted molecular mass of Ndfip1 protein. Quantification analysis showed that the protein levels of Ndfip1 were significantly decreased in the cortex and hippocampus in APP/PS1 mice, compared to these from WT controls (Figure 3). The expression level of DMT1 was consistent with our previous reports (Zheng et al., 2009). Both DMT1-IRE and DMT1-nonIRE were significantly elevated in the cortex and hippocampus in APP/PS1 mouse brain (Figure 3).

### The Distribution and Expression of Ndfip1 in APPsw Transfected SH-SY5Y Cells

**Figure 4:**
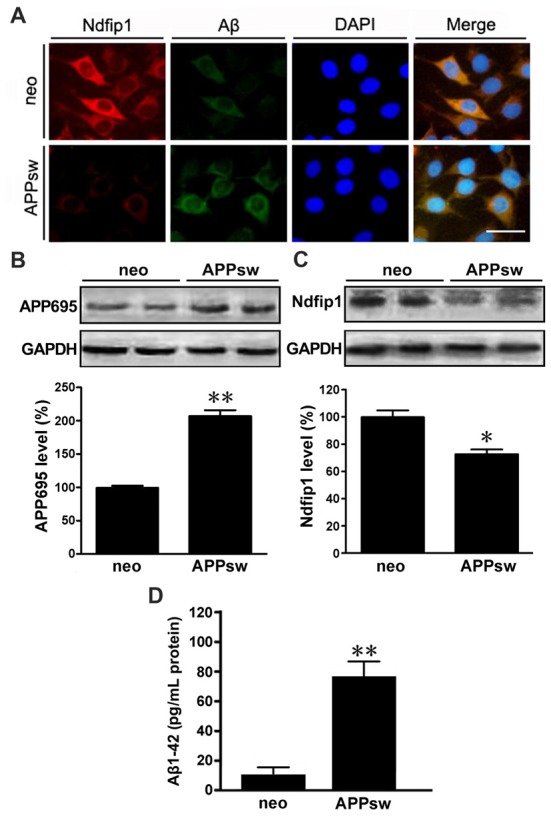
Distribution and expression of Ndfip1 in APPsw transfected SH-SY5Y cells. Immunofluorescence staining showing that Ndfip1 (red) and Aβ (green) overlapped in APPsw cells and neo cells **(A)**. Scale bar = 50 μm. APPsw cells showed significantly increased levels of APP695 **(B)** and decreased levels of Ndfip1 **(C)**, compared with neo cells. ELISA analysis showed a significant increased level of Aβ1–42 peptide in the conditioned medium of APPsw transfected SH-SY5Y cells (**D**; *n* = 3; *P < 0.05, **P < 0.01).

We next examined the distribution and expression of Ndfip1 in APPsw transfected SH-SY5Y cells *in vitro*. Immunofluorescence double-labeling showed that with Ndfip1 and Aβ overlapped in both APPsw and neo cells. However, APPsw cells showed a lower immunofluorescence staining for Ndfip1 accompanied by a higher immunofluorescence for Aβ, compared with neo cells (Figure 4A).

Cell lysate samples from both APPsw and neo cells were analyzed for the protein levels of APP695 and Ndfip1. Immunoblotting for APP695 protein revealed a major band at 86 kDa. Consistent to the results from vivo experiments, the protein level of Ndfip1 was decreased, and APP695 was increased in APPsw cells, as shown in Figures 4B,C.

ELISA analysis was performed to assess the Aβ1–42 level in the conditional medium of APPsw and neo cells. The level of Aβ1–42 peptide was increased in APPsw cells, as shown in Figure 4D.

### Transfection of Ndfip1 Down-Regulated the Expression Levels of DMT1 in APPsw Cells

**Figure 5:**
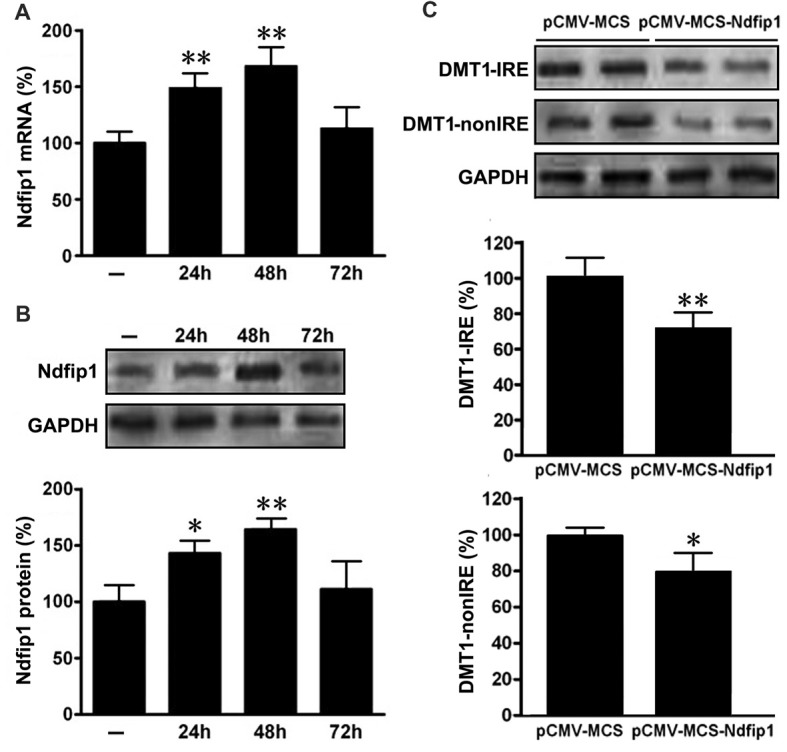
Expression of Ndfip1 and DMT1 in APPsw cells transfected by Ndfip1. Both mRNA **(A)** and protein levels **(B)** of Ndfip1 were significantly increased after transfected with Ndfip1 (pCMV-MSC-Ndfip1) for 24 and 48 h, compared with vector controls (pCMV-MSC). The protein levels of two isoforms of DMT1, DMT1-IRE and DMT1-nonIRE, were markedly reduced after Ndfip1 transfection for 48 h (**C**, *n* = 3; *P < 0.05, **P < 0.01).

In order to determine whether Ndfip1 is involved in DMT1 degradation *in vitro*, APPsw cells were transfected with pCMV-MCS-Ndfip1 and vector control, respectively. The transfection efficiency was evaluated with real time PCR and Western blot. As shown in Figure 5, both mRNA and protein levels of Ndfip1 were significantly increased after transfected with Ndfip1 plasmid for 24 and 48 h.

We then detected the protein levels of two isoforms of DMT1, DMT1-IRE and DMT1-nonIRE, after Ndfip1 transfection for 48 h. Immunoblotting results showed that the expression levels of both DMT1-IRE and DMT1-nonIRE in Ndfip1 transfected APPsw cells were significantly decreased, compared to these from vector controls (Figure 5C).

### Overexpression of Ndfip1 Reduced Iron Influx, APP695 Level and Aβ Secretion in APPsw Cells

**Figure 6:**
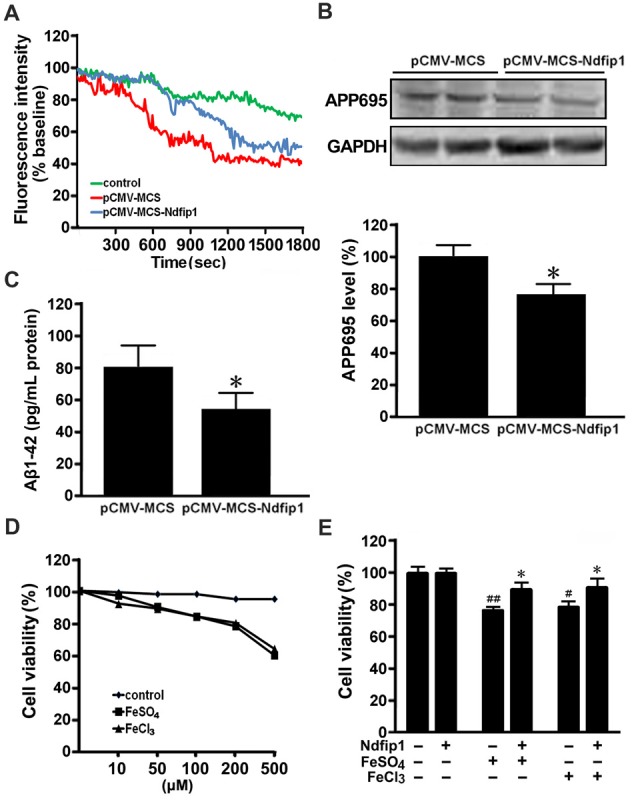
Overexpressed Ndfip1 reduced iron influx, APP695 level, Aβ generation and inhibited cell death. There was a significant increase in calcein fluorescence intensity in Ndfip1 transfected APPsw cells (pCMV-MSC-Ndfip1), compared with vector controls (pCMV-MSC; **A**). Western blot showed a significant decreased level of APP695 in the Ndfip1 transfected APPsw cells **(B)**. ELISA analysis showed a significant decreased level of Aβ1–42 peptide in the conditioned medium of Ndfip1 transfected APPsw cells **(C)**. MTT assay confirmed that 200 μM FeSO_4_ or FeCl_3_ were selected to treat the Ndfip1 transfected APPsw cells **(D)**. Cell viability analyses showed that iron-treatments significantly reduced the cell viability. Conversely, overexpressed Ndfip1 inhibited iron-induced cell damage (**E**; *n* = 3; *P < 0.05, compared with vector controls with iron treatments; ^#^*P* < 0.05, ^##^*P* < 0.01, compared with vector controls).

Our previous studies have demonstrated that DMT1 up-regulation is involved in APP processing and Aβ generation through promoting iron accumulation (Zheng et al., 2009). To further verify whether overexpression of Ndfip1 could reduce the iron influx into the nerve cells, ferrous uptake was detected quenching of calcein fluorescence, which is an indicator of intracellular iron level. After Ndfip1 transfected for 48 h, APPsw cells were incubated with 0.5 μM calcein AM, harvested and analyzed in cuvettes. After stabilization of the fluorescence signal, 100 μM ferrous sulfate (final concentration) was added to the cuvettes. As shown in Figure 6A, ferrous sulfate quenched calcein fluorescence in a time-dependent manner. Notably, the fluorescence intensity in Ndfip1 transfected cells was stronger than that in control vectors.

Western blot was performed to assess the APP695 level in the cell lysate samples from Ndfip1 transfected APPsw cells and vector cells. Overexpression of Ndfip1 lead to a decreased APP695 level, as compared with vector controls (Figure 6B).

ELISA analysis was performed to assess the Aβ1–42 level in the conditional medium of Ndfip1 transfected APPsw cells and vector cells. Overexpression of Ndfip1 lead to a decreased level of Aβ1–42 peptide, as compared with vector controls (Figure 6C). Taken together, these results indicate that overexpression of Ndfip1 reduced DMT1-mediated ferrous iron uptake and hence inhibited Aβ generation in APPsw cells.

### Overexpression of Ndfip1 Attenuated Iron-Induced Cell Damage in APPsw Cells

To evaluate the role of Ndfip1 in protecting against iron-induced cell death, we measured the cell viability in iron-treated APPsw cells transfected with Ndfip1. According to cell viability screening, both ferrous sulfate (FeSO_4_) and ferric chloride (FeCl_3_) at the concentration of 200 μM were chosen to treat the Ndfip1 transfected APPsw cells for 48 h (Figure 6D). MTT analysis revealed that iron-treatments significantly reduced the cell viabilities, whereas Ndfip1 overexpression reversed iron-induced cell damage (Figure 6E).

## Discussion

Although iron is an essential nutrient element for a variety of cellular biological processes, excessive intracellular iron can cause oxidative stress, protein aggregation and cell death (Hadzhieva et al., 2014). Several studies have shown that the iron content is abnormally high in AD brain, suggesting that accumulation of iron is involved in the pathogenesis of AD (Goodman, 1953; García de Ancos et al., 1993; Mantyh et al., 1993; Smith et al., 1997; Yamamoto et al., 2002; Nakamura et al., 2007; Bousejra-ElGarah et al., 2011). Since iron ions cannot freely pass through the plasma membrane, several metal transporters, such as DMT1, are essential for maintaining cellular iron homeostasis (Gunshin et al., 1997; Burdo et al., 2001, 2004; Simpson et al., 2015). We have previously reported that high expression levels and changes in distribution of DMT1 have been found in postmortem AD and APP/PS1 mouse brain, suggesting that DMT1 might be involved in iron dyshomeostasis as well as in Aβ generation and deposition (Zheng et al., 2009). Interestingly, recent studies have shown that Ndfip1 regulates DMT1 degradation through ubiquitination pathway, downregulates DMT1 expression and activity, reduces the accumulation of intracellular iron (Foot et al., 2008, 2011), and prevents metal toxicity in human neurons (Howitt et al., 2009). However, whether Ndfip1 is associated with AD progression through mediating DMT1 degradation and ubiquitination has not been fully understood. In this study, we found that the protein level of Ndfip1 was decreased, but DMT1 was increased significantly in APP/PS1 mouse brain. Importantly, overexpression of Ndfip1 could reduce DMT1 expression, iron influx and Aβ secretion *in vitro*. The present results indicate that low level of Ndfip1 in the brain might be involved in AD progression, through decreasing DMT1 ubiquitinated degradation and increasing iron accumulation in the brain.

The present study has shown that Ndfip1 immunoreactivity is predominantly located in neuronal somata and Aβ-positive plaques in APP/PS1 mouse brain. Further, a significant low level of Ndfip1 is detected in APP/PS1 mice and APPsw overexpressed SH-SY5Y cells. Within the same mouse and cell models, we have previously reported that DMT1 is also found in amyloid plaques, and the expression level is elevated in the mouse brain and APPsw cells (Zheng et al., 2009). Since DMT1 is a Ndfip1 target protein, the similar distribution and the opposite expression trend of Ndfip1 and DMT1 suggest that low level of Ndfip1 is responsible for the elevation of DMT1 in AD brain. We then analyzed whether overexpression of Ndfip1 could enhance DMT1 degradation by using Ndfip1 transfected APPsw cells. Accompanied by an increased level of Ndfip1, the protein levels of both DMT1-IRE and DMT1-nonIRE are decreased dramatically. Our results are consistent with previous studies in PD models, Ndfip1 is decreased in 6-OHDA-treated rats and MES23.5 cells, whereas Ndfip1 overexpression leads to a decrease in DMT1 level *in vitro* (Howitt et al., 2014; Jia et al., 2015; Liu et al., 2015; Xing et al., 2016).

We have previously reported that treatment with iron in drinking water increases APP protein expression and phosphorylation, enhances amyloidogenic APP cleavage and Aβ deposition in APP/PS1 mouse brain (Guo et al., 2013a,b). RNAi silencing of DMT1 reduces iron influx and attenuates iron-induced amyloidogenic APP processing *in vitro* (Zheng et al., 2009). In this study, we found that overexpression of Ndfip1 could reduce iron influx and Aβ1–42 peptide, suggesting that increase in expression level of Ndfip1 degrades DMT1 and then inhibits Aβ generation by reducing DMT1-mediated iron influx. Iron accumulation not only participates in Aβ generation, but also involved in neuronal death in the pathogenesis process of AD (Zheng et al., 2009; Guo et al., 2013a,b; Zhang et al., 2018). We then evaluated whether Ndfip1 played a role in protecting against iron-induced cell injury, and the results revealed that excessive iron aggravated neuronal injury, whereas Ndfip1 overexpression reversed iron-induced cell damage. Several studies have demonstrated that reduction of brain iron accumulation is a potential strategy for AD prevention (Zheng et al., 2009; Guo et al., 2013a,b; Zhang et al., 2018; Zhao et al., 2018). However, further studies are needed to evaluate whether regulation of brain iron homeostasis through enhancing Ndfip1 protein level is benefit for blocking the neuropathological process of AD.

In conclusion, the present results outline the significant role of Ndfip1 in correcting iron abnormalities, inhibiting Aβ secretion and protecting against iron-induced neuronal injure by regulating DMT1 degradation. Therefore, Ndfip1 might be a potential molecular target for AD prevention and treatment.

## Author Contributions

Z-YW and WZ conceived and designed the study. JT, WZ, X-LL and Y-HC performed the experiments and data analyses. JT drafted the manuscript. Z-YW and WZ wrote the article. All authors read and approved the manuscript.

## Conflict of Interest Statement

The authors declare that the research was conducted in the absence of any commercial or financial relationships that could be construed as a potential conflict of interest.
